# Prototype of Robotic Device for Mobility Assistance for the Elderly in Urban Environments

**DOI:** 10.3390/s20113056

**Published:** 2020-05-28

**Authors:** Daniel Leite, Karla Figueiredo, Marley Vellasco

**Affiliations:** 1 Department of Electrical Engineering, Pontifical Catholic University of Rio de Janeiro (PUC-Rio), 22451-900 Rio de Janeiro, Brazil; daniels.leite@hotmail.com; 2 Department of Informatics and Computer Science, Institute of Mathematics and Statistics, State University of Rio de Janeiro (UERJ), 20550-900 Rio de Janeiro, Brazil; karlafigueiredo@ime.uerj.br

**Keywords:** robotic rollator walker, fuzzy logic, autonomous navigation, outdoor environment, elderly

## Abstract

This study aims to develop a prototype of an autonomous robotic device to assist the locomotion of the elderly in urban environments. Among the achievements presented are the control techniques used for autonomous navigation and the software tools and hardware applied in the prototype. This is an extension of a previous work, in which part of the navigation algorithm was developed and validated in a simulated environment. In this extension, the real prototype is controlled by an algorithm based on fuzzy logic to obtain standalone and more-natural navigation for the user of the device. The robotic device is intended to guide an elderly person in an urban environment autonomously, although it also has a manual navigation mode. Therefore, the device should be able to navigate smoothly without sudden manoeuvres and should respect the locomotion time of the user. Furthermore, because of the proposed environment, the device should be able to navigate in an unknown and unstructured environment. The results reveal that this prototype achieves the proposed objective, demonstrating adequate behaviour for navigation in an unknown environment and fundamental safety characteristics to assist the elderly.

## 1. Introduction

Ageing of the population, which is already a reality in developed countries, has become increasingly present in developing countries, such as Brazil. According to data from the Brazilian Institute of Geography and Statistics (IBGE) [1,2,3], projections indicate that, in 2050, the elderly population of Brazil will be approximately 25% of its population. In an interview with the São Paulo State Research Support Foundation (FAPESP) [4], Alexandre Kalache, a doctor and public health researcher, warned about the need for public policies aimed at this new reality, citing the case of France. France took 115 years to double the proportion of the elderly from 7% to 14%, while Brazil’s elderly population is expected to double from 9% to 18% in just 17 years.

In a guide published by the World Health Organization [5], several characteristics that a city must have to meet the needs of an elderly population are described. Among them, the city should be adapted to mobility needs, such as wide sidewalks, low steps, proper signage, and smooth, level, non-slip surface pavements. However, even in an adapted city, part of the elderly population makes use of locomotion assistance devices, such as walking sticks and walkers, because they may have some motor, cognitive, or visual impairment, or may desire greater comfort and safety when moving around.

With a maturing world population, the search for new technologies and methods that help in improving the quality of life of the elderly population has been one of the focuses of the scientific community. Among the research themes is the study of mobility assistance devices for people with cognitive or visual difficulties. In a study by Di et al. [6], for example, a robotic cane composed of an omnidirectional mobile robot was developed. The objective was to assist in maintaining an adequate posture, preventing falls, and indoor locomotion. This structure is similar to that proposed by Dubowsky et al. [7]. However, the base of the cane is differential, i.e., it has two parallel wheels with independent actuations, and the rod is attached to the base rigidly, with a force/torque sensor at the junction rod/base.

Among the functional objectives of the two projects, the main differences are that, in the project reported by Di et al. [6], the maintenance of the posture and balance of the user, which was the main objective, was achieved through the actuators connected at the rod/base junction. Regarding the project of Dubowsky et al. [7], the posture and balance of the user were provided by the design of the cane, i.e., passively. The main objectives were to guide, locate, and monitor users moving in closed environments, such as rest clinics and health homes, preventing the elderly from encountering obstacles that may lead to falls.

Another type of structure widely used is walker-based. In a study by Wasson et al. [8], for example, a commercial walker was used as a structure. In the projects of Zhou et al. [9], Hirata et al. [10], Taghvaei et al. [11], and Ohnuma et al. [12], they also used walker-based structures. Despite their similar structures, these projects used different methodologies and approaches to act in the assistance of locomotion and fall prevention.

Wasson et al. [8] proposed, among other objectives, regulating the navigation autonomy of the device based on the navigation intention of the user. The intention is estimated from the data of the force/torque sensors, present in the handles of the walker, about the obstacles captured by the distance sensors and the history of actuations. As a result, the walker offers resistance to locomotion for paths that differ from the autonomous navigation of the device, i.e., that are not considered safe. Zhou et al. [9] had a very similar approach to that of Wasson et al. [8], differing from the latter because the walker was equipped with a Global Positioning System (GPS) sensor for locomotion outdoors. In the studies by Hirata et al. [10] and Taghvaei et al. [11], the focus was on the prevention of falls from the identification and classification of the conditions of falls and walking. In these studies, infrared sensors and cameras were used, respectively, in addition to force/torque sensors, to classify the posture of the user as a “fall posture”, with its subtypes, or “walk”, from which the device acts to provide the necessary support to the classified condition.

Unlike the projects presented above, Ohnuma et al. [12] used a structure involving the user, with a design that provided greater support. The objective of this project was the implementation of an autonomous navigation control that reacted in a natural and smooth way when the person was walking. Infrared sensors were used to observe the movement of the feet and hip of the user, and a particle filter (Thrun et al. [13]) was applied to estimate the displacement of the user, based on the sensor data. Therefore, the movement of the device was based on the prediction of the user displacement. Other works related to fall prevention and physical assistance robots for the elderly population can be observed in [14,15,16].

In the above research, it can be observed that, regardless of the type of structure proposed for the device or the type of support it offers to help the posture of the user, it is crucial that the autonomous navigation occurs smoothly, without “competing” directly with the intention of the user to move. Otherwise, the device itself may cause imbalance, or, in the worst case, the fall of the elderly person.

To detect the navigation intention of the user many approaches can be taken, e.g., using forces and torque sensors, as used for Wasson et al. [8], or less invasive one’s that uses vision and laser sensors, to estimate the intention of the user, like the work of Ohnuma et al. [12]. In a more controlled environment, like hospital or nursing homes, techniques that related visual attention and eye-hand coordination, [17,18], could also be used as an interface to users who have a more severe motor disability in their arms.

In this study, a prototype to assist the locomotion of the elderly in urban environments was developed. The unknown and unstructured environment proposed for navigation imposes a significant challenge for the prototype, in addition to being different from those in the works presented above. Another difference is the use of a fuzzy-logic-based navigation algorithm. The choice of this computational intelligence technique was made because it enables the translation of behaviours and situations into a small set of rules that extrapolate to a more general behaviour.

For the initial prototype, the focus was on the navigability of the device; therefore, aspects, such as stability, ergonomics, and support, will be covered in the next prototypes. The initial characteristics of the prototype, as well as some aspects of its functionalities, were established based on the related works and the work conducted by Rivero et al. [19,20,21], who employed the quality-by-design technique [22,23,24] in the development of their prototype.

The rest of the work is divided into three sections. In Section 2, the details of the electronic parts and autonomous navigation based on fuzzy logic, as well as a manual for the developed device, are presented. In Section 3, the tests and results are discussed. In Section 4, the conclusions are provided, and directions for future work are described.

## 2. Materials and Methods

In this section, the functional aspects of the device, as well as its structural characteristics, electronics (drivers, sensors, actuators, etc.), and software, are addressed. The differences between the fuzzy inference system of the virtual mode [25] and the built prototype, aiming at autonomous navigation of the robotic device, are also presented.

### 2.1. Functionality and Structure

Based on the data obtained from the work of Rivero et al. [19,20,21], some functionalities and boundary conditions were established, such as the weight, speed, and size of the prototype. Based on these data, the mechanical and electronic components used were specified. The compilation of recommendations obtained from the work of Rivero et al. [19,20,21] is presented in Table 1.

**Figure 1:**
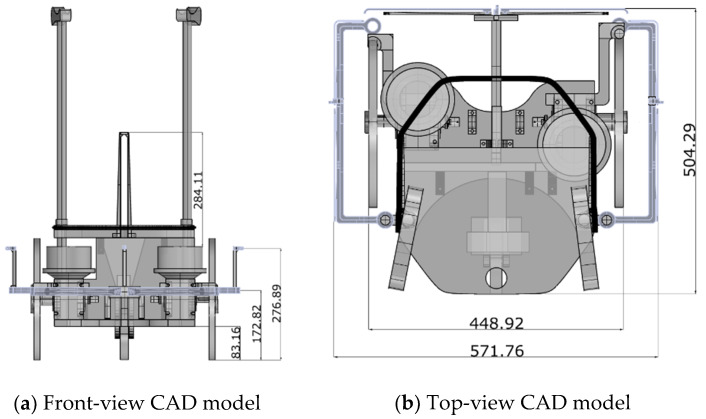

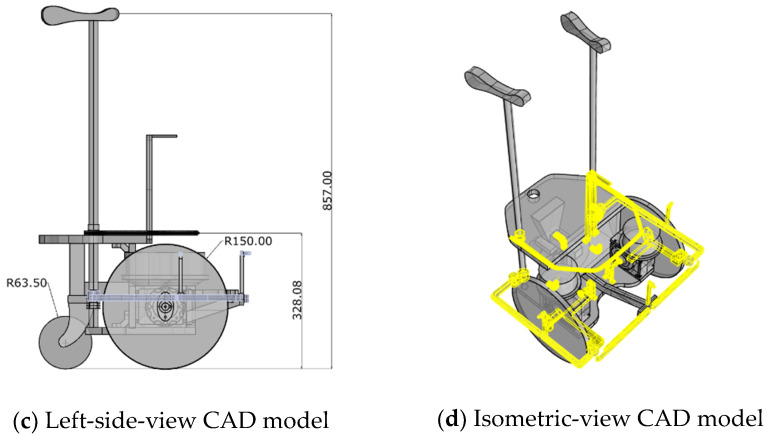
Computer-aided design (CAD) views of the prototype (dimensions in millimetres).

In the development of the device, a structure based on a walker, where the base is a differential mobile robot with a beaver wheel for support, was considered. The walker-like design was adopted because it offered greater stability to the user in comparison with the cane-like structure. Additionally, the differential robot architecture was adopted because it enabled simpler construction and actuation in comparison with the omnidirectional structure. On the other hand, the omnidirectional structure has the advantage of providing greater manoeuvrability to the device. Another advantage of the adoption of a differential structure, in relation to the omnidirectional one, is that its parallel wheels facilitate the transposition of small obstacles, such as gaps between sidewalks. The computer-aided design (CAD) model with the dimensions, in millimetres, of the prototype is illustrated in Figure 1.

The material used for the construction of the prototype was mostly wood, of the plywood type and medium-density fibreboard. The parts that required greater design accuracy, such as the sensor fittings, were designed and then produced in acrylonitrile butadiene styrene plastic using a 3D printer. In Figure 1d, the printed pieces are highlighted in yellow. The choice of materials was based only on the practicality and ease of handling for construction. Thus, at this moment, the prototype does not possess the necessary resistance to operate safely with an elderly person; however, it is sufficiently strong to perform navigation and locomotion tests that can validate the navigation control model in unknown environments.

### 2.2. Actuators and Sensors

To provide the necessary traction to the system, two sets of Bosch DC GPB F006 KM0 60N [33] motors coupled to MKS R4 1:10 63 B14 [34] reduction boxes were used, one set for each wheel. This motor–reducer assembly gave the system an open rated rotation of approximately 250 RPM and a nominal torque per set of 39 Nm. Each of the engine outputs was coupled with an optical incremental encoder, Bourns ENA1J-B28-L00100L [35], to feedback the motor speed control circuit.

A proportional–integral–derivative (PID) controller, whose set of applied gains can be observed in Table 2, performed the rotation control of each motor, and the power was generated through a Pololu Dual VNH5019 driver [36].

**Figure 2:**
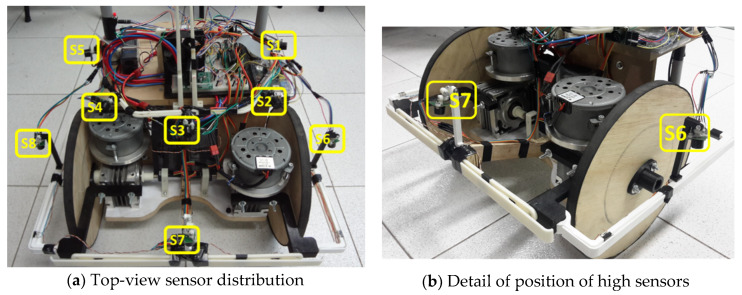
Distribution of ultrasonic sensor at the prototype.

For the device to detect the obstacles that could lead to its collision or the fall of a user, ultrasound distance sensors were used. This type of sensor was applied because of its low cost and immunity to noise owing to the incidence of infrared-wavelength light. These characteristics influenced the choice of sensor because of the type of environment proposed for navigation, i.e., external environments. The sensor chosen was the MaxBotix LV-MaxSonar EZ1 sensor [37], purchased on the Internet and delivered to Rio de Janeiro, Brazil. In total, eight sensors were used, whose distribution in the prototype is illustrated in Figure 2.

Sensors S1–S5 were used to detect collision obstacles, while S6–S8 were applied to detect unevenness, such as holes in the sidewalk or a curb. For the S6–S8 sensors to measure height information, their positioning was directed to the ground, but with an inclination that would allow anticipating the presence of unevenness without suffering interference from the device structure itself, as in Figure 2b.

The activation of these eight sensors was performed in an interleaved manner, between sensors that point to close directions, to avoid interference resulting from crosstalk. In the adopted drive sequence, the S2 and S8 sensors are actuated simultaneously. After completing the reading, the S2 sensor triggers a command to the beginning of the reading of the S4 and S6 sensors. When the reading is complete, the S4 sensor sends a reading command to the S1, S5, and S7 sensors. With the S7 sensor reading complete, it sends a reading command to the S3 sensor, completing a reading cycle. This drive sequence was adopted so that sensors that point in the same direction do not perform simultaneous readings, thus avoiding the occurrence of the crosstalk effect.

Another procedure, adopted to improve the accuracy of the distance measurement obtained by the sensors, was to apply a filter to each measurement performed. The filter exerts an effect similar to a moving average; however, its activation occurs only when the value measured by a given sensor has a variation of 50% compared with the previous measure. In Equations (1) and (2), the implementation of the filter is presented.

(1) $$\mathrm{If}\;\Delta_{distance}>50\%\;\{\begin{array}{c} {\mathrm{Distance}}_{used}=\left(\frac{{\mathrm{Distance}}_{read}+2\times {\mathrm{Distance}}_{old}}{3}\right)\\ {\mathrm{Distance}}_{old}=\left(\frac{{\mathrm{Distance}}_{read}+{\mathrm{Distance}}_{old}}{2}\right) \end{array}$$

(2) $$Else\;\{\begin{array}{c} {\mathrm{Distance}}_{used}={\;\mathrm{Distance}}_{read}\;\\ {\mathrm{Distance}}_{old}={\;\mathrm{Distance}}_{read} \end{array}$$

**Figure 3:**
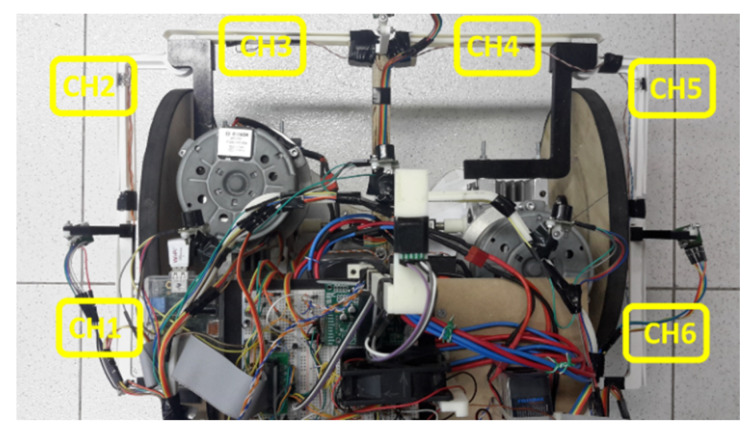
Distribution of contact switches.

In addition to the distance sensors, the system, for safety reasons, still contains contact switches to identify any collision of the device. As illustrated in Figure 3, switches CH1–CH6 are scattered around the prototype and detect collisions that may occur at the front, right side, and left side of the prototype, thereby increasing the safety of the system. The information from the left side switches, associated with CH1 and CH2 will be treated as only one information, called Bumper1 (Bp1), i.e., the switches are connected in are in series to an input of the system. The same procedure will adopt to front switch, CH3 and CH4, called Bumer2 (Bp2), and right-side switches, CH5 and CH6, called Bumper3 (Bp3).

**Figure 4:**
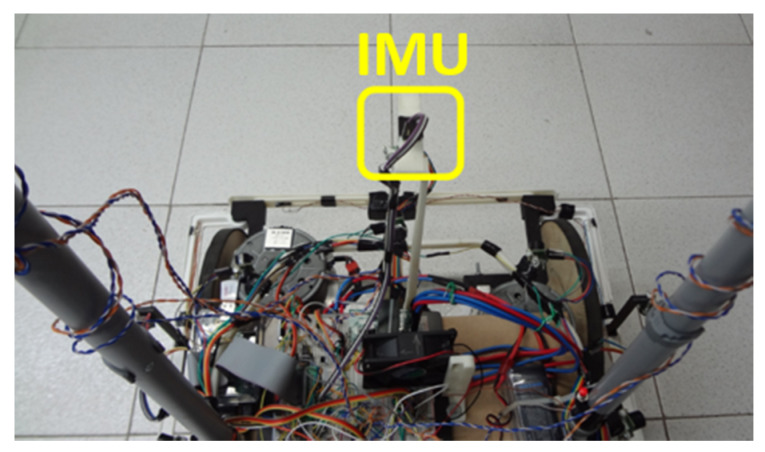
Inertial measure unit (IMU) sensor in prototype.

A low-cost inertial measure unit (IMU) was used to identify the orientation of the device. The chosen IMU was the Pololu MinIMU-9 v2 [38], purchased on the Internet and delivered to Rio de Janeiro, Brazil, and its positioning in the prototype can be observed in Figure 4.

#### 2.2.1. Manual Drive

**Figure 5:**
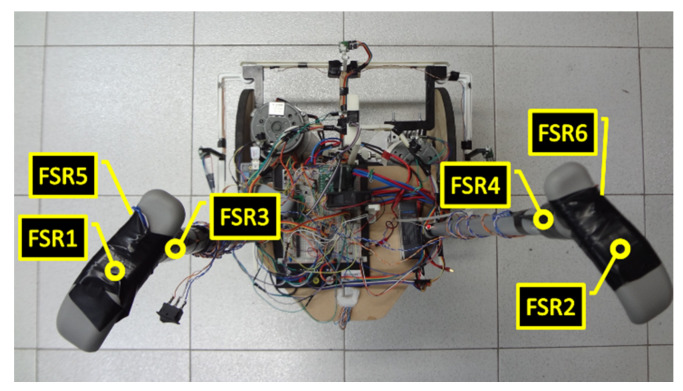
Distribution of FSR 400 sensor at the prototype.

To detect the action of the user on the device, FSR 400 force sensors [39] were used. This type of sensor was chosen because of its low cost, particularly when compared with torque sensors. In total, six FSR 400 sensors were used. These sensors were positioned on the handle of the device, as depicted in Figure 5. The force sensors identified as FSR1 and FSR2 are responsible for detecting whether the user is holding the device with both hands. If one of the sensors does not capture a minimum force of the user, the operation of the engines is interrupted and blocked. This value of the minimum force required is obtained empirically by recording the sensor reading when the user holds the prototype handle firmly.

The FSR3 and FSR4 sensors, shown in Figure 5, are the interfaces for direct user actuation on the left and right motors, respectively. Therefore, if the user exerts a force above a threshold on one of the actuation sensors, a velocity value proportional to the perceived force above this threshold is sent to the corresponding motor. The same is true for the FSR5 and FSR6 force sensors; however, their actuation causes the motors to rotate in the opposite direction to that of the FSR3 and FSR4 sensors. The value of the threshold of sensors FSR3–FSR6, as well as of the threshold for FSR1 and FSR2 force sensors, are empirically obtained. The absolute maximum speed driving for FSR3–FSR6 sensors is of 2.5 rad/s for the motor corresponding to the actuation. This process of engine activation, in both directions, through force sensors is the user interface for the manual operation of the device.

The action of the user takes priority over the fuzzy inference system (FIS), described in Section 2.4. Therefore, while the user is acting on the engine—that is, applying a force on sensors FSR 3, FSR4, FSR5, or FSR6, as depicted in Figure 5—the values provided by the FIS are ignored.

### 2.3. Interfaces of Hardware and Software

The processor hardware used was the single-board computer (SBC) Raspberry Pi model B Rev2 [40], along with MCP3008, Analogue-to-Digital (A/D) converter, [41] and MCP23017, I/O expander, [42] chips to expand the input and output ports. This SBC was used because of its processing power and large developer community, as well as its Linux-based operating system. The last feature was necessary because the virtual mode [25] was developed using the robotics framework Player [43,44], which required a Linux-based operating system for its installation. The objective of using this framework was the integration between the virtual and real models, enabling the use of the same codes developed in the virtual platform without requiring significant changes.

Programs that access Player server interfaces are called client programs. In this work, two client programs were used. The main program, developed in this work, which accesses the Player server interfaces, runs the FIS and performs autonomous or manual navigation. The navigation application uses a Google Maps application programming interface (API) to trace the navigation routes on foot and sends information about the distance and direction of the objective to the Player server.

**Figure 6:**
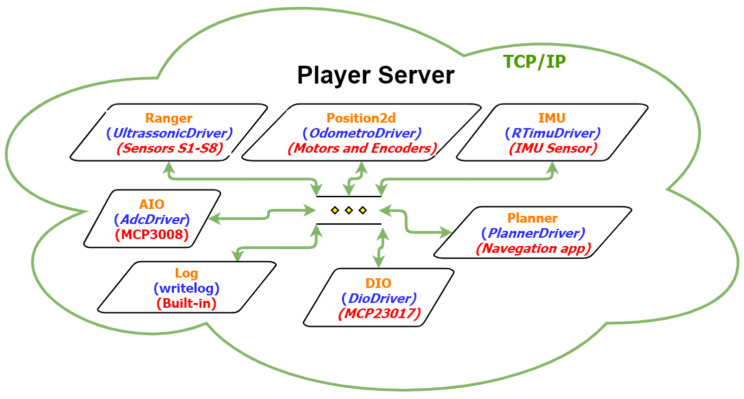
Macro diagram of Player server drivers.

To integrate the Player platform with the real prototype, it was necessary to program the drivers for each sensor and actuator. In Figure 6 is shown a macro representation of Player server drivers interconnection, where the cloud represents the Player server, the green line the bidirectional communication in TPC/IP protocol and each box a written driver of the prototype. In each driver, you can see in orange the name of Player interface that was used, in blue the name of the driver and in red the hardware associated with the driver. In total, seven drivers were used: the *UltrassonicDriver*, to manipulate the data from ultrasonic sensors (Figure 2); the *OdometroDriver*, to control de speed of each DC motor; the *RTimuDrive*, to read the data from IMU sensor (Figure 4); the *AdcDriver*, to get the dada from MCP3008, A/D converter chip; the *PlannerDriver*, to receive the data from navigation app; *writelog*, to log the robot data; and *DioDriver*, to manipulate de DIO from MCP23017, I/O expander chip. The developed drivers and diagrams of the associated hardware, as well as the execution flowchart of the main client program, are described in the following subsections.

#### 2.3.1. AdcDriver

AdcDriver is designed to encapsulate the data provided by the MCP3008 chip, (A/D) converter, within the Analog I/O (AIO) Player server interface. The AIO interface is responsible for managing the analogue inputs and outputs of the Player server, providing functions for reading and writing them. In the AdcDriver case, because the analogue-to-digital (A/D) chip used has only inputs, the driver does not operate the writing functions of the analogue outputs.

**Figure 7:**
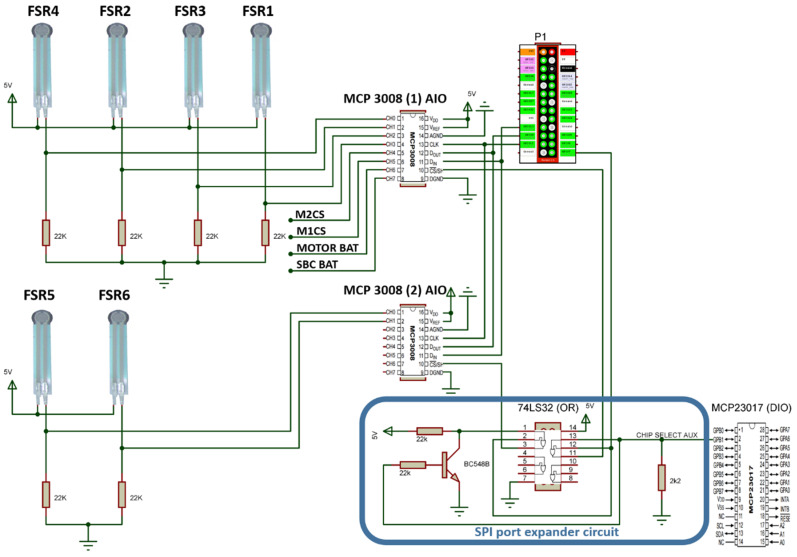
Hardware diagram associated with AdcDriver.

This driver processes messages sent by client programs to read the values of analogue ports from MCP3008 (1) and (2), and the reading frequency of these ports is 20 Hz. This driver is used to read the battery voltage levels from motors and the SBC, MOTOR BAT and SBC BAT, motors driver current sensors, M1CS and M2CS, and the FSR1-FSR6 sensors (Section 2.2.1). The hardware diagram associated with this driver can be seen in Figure 7. In addition, Figure 7 also shows the P1, SBC IO header 1 and SPI port expander circuit, the logic port OR and the transistor used to expand the possibilities of Raspberry PI SPI bus interface from two to three devices.

#### 2.3.2. DioDriver

DioDriver was designed to manage the input and output ports of the MCP23017, I/O expander, chip and encapsulated them in the Digital I/O (DIO) interface of the Player server. This driver processes messages from client programs to read the input ports and change the logical state of the output ports.

**Figure 8:**
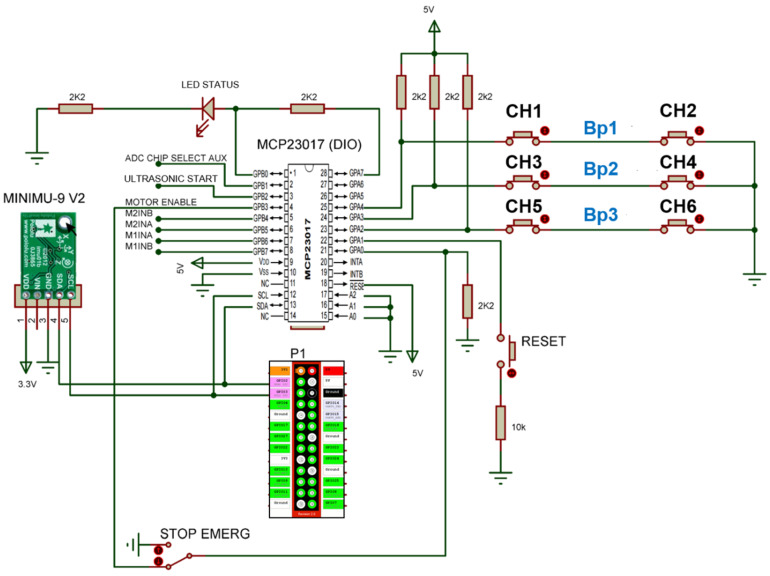
Electrical diagram of components associated with DioDriver.

The driver is used as input of the contact switch, CH1–CH6 combined to make the Bp1–Bp3 status Figure 3 of Section 2.2, which identifies collisions, and to generate client program execution status, LED of status, and emergency stop command return. The processing of this driver occurs at a frequency of 20 Hz, and the electrical diagram can be observed in Figure 8.

In addition to serving the DioDriver, the MCP23017, I/O expander, is also used by other drives, such as input or output ports, like the start command to read from ultrasonic sensors and as ports of command and control from the DC motors power driver. Figure 8 also shows the electronic connection from the IMU sensor, Figure 4 of Section 2.2, identified as MINIUM-9 V2.

#### 2.3.3. RTimuDriver

RTimuDriver was designed to manage data from the inertial measure unit MinIMU-9, Figure 4 of Section 2.2, and encapsulate them in the IMU interface of the Player server. Through the driver, it is possible to configure the format in which the IMU data are published, i.e., Euler angles, quaternion, sensor data without fusion, or position and orientation information. The frequency with which the IMU data are published is 20 Hz, and the electrical diagram of the sensor connection can also be observed in Figure 8, identified as element MINIUMU-9 v2.

#### 2.3.4. UltrasonicDriver

**Figure 9:**
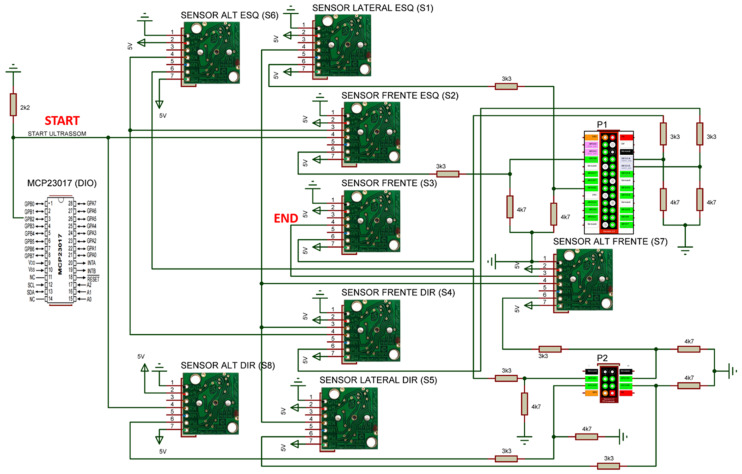
Interconnection diagram of ultrasound sensors.

UltrasonicDriver was designed to obtain data from ultrasound sensors and encapsulate them in the Player server Ranger interface. How often data are published depends on the number of independent readings and the desired value for the average of the readings. This rate is because each independent reading adds a wait of 50 ms, which is the time of the reading cycle of a sensor. In the application of this work, four sets of sensors are read—that is, four independent readings—and the driver do perform the data average of sensors reading. Therefore, the frequency with which the data are published is 5 Hz. Figure 9 shows the connection diagram of these sensors.

#### 2.3.5. OdometroDriver

**Figure 10:**
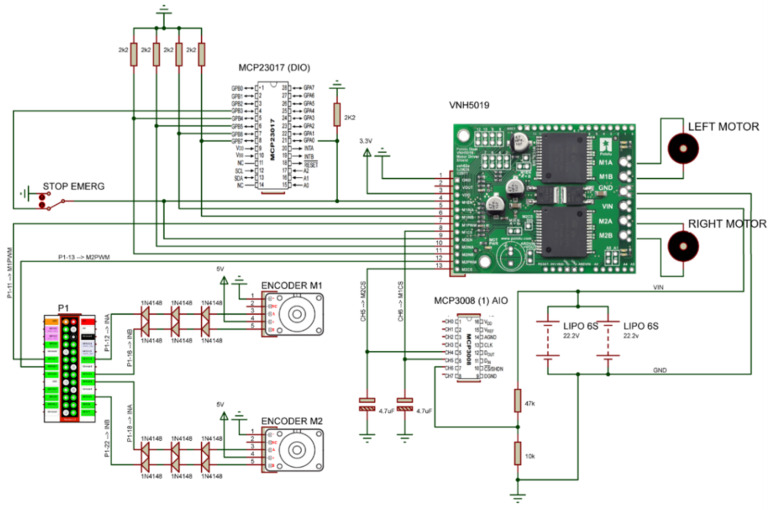
Diagram of the motor actuation and control circuit.

The OdometroDriver was created to perform the control of the angular velocity of each engine. This driver reads the rotation of each engine through the encoders and sends the actuation commands to the power driver. The data from the encoders and power driver are encapsulated in the position2d interface of the Player server. This driver also instantiates independent threads for the routines of the PID controls of each engine. Figure 10 illustrates the electrical diagram of the elements related to this driver.

#### 2.3.6. PlannerDriver

**Figure 11:**
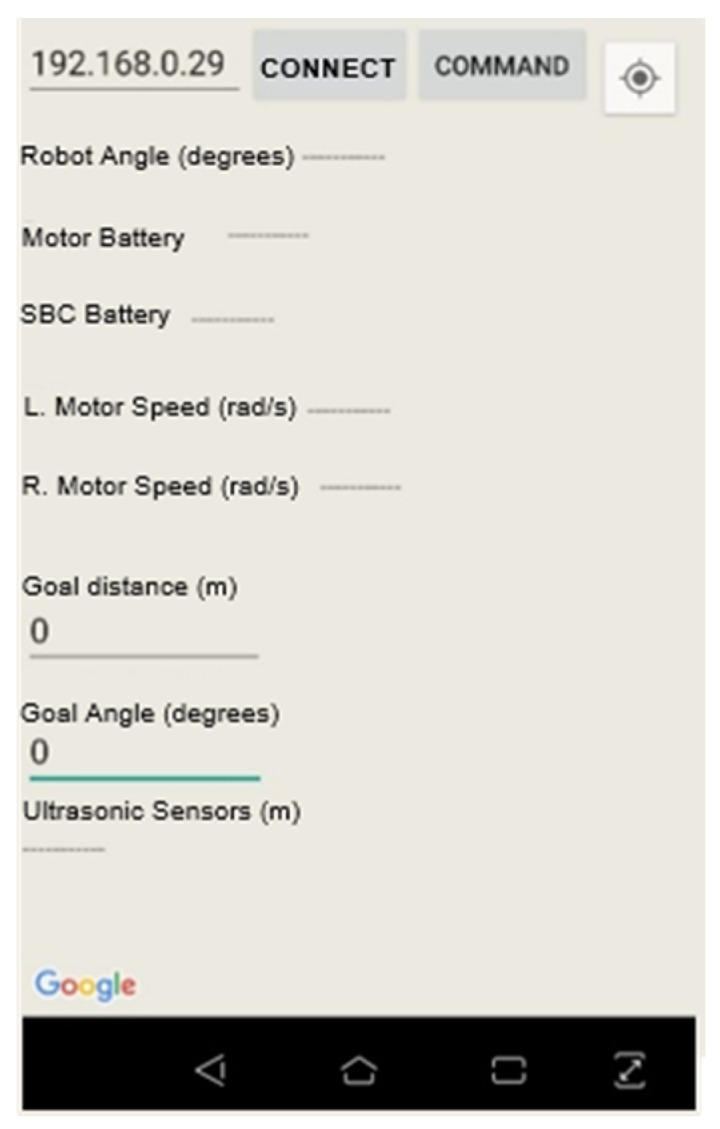
Android application screen for standalone navigation test.

PlannerDriver was used as an interface between the navigation application and the robotic device. The Java Client 3 [45] library was included in the navigation application to act as a player server client, having access to all drivers that have been instantiated on the server. To perform the first autonomous navigation tests, a simpler version of navigation application was created, whose screen can be observed in Figure 11. In this simplified version, the user manually inserts information regarding the distance and goal orientation, simulating the results of the final application autonomously through the Google Maps API route system. In this manner, it was possible to perform tests in places where there were no navigation routes or GPS signals.

#### 2.3.7. Main Client Program

**Figure 12:**
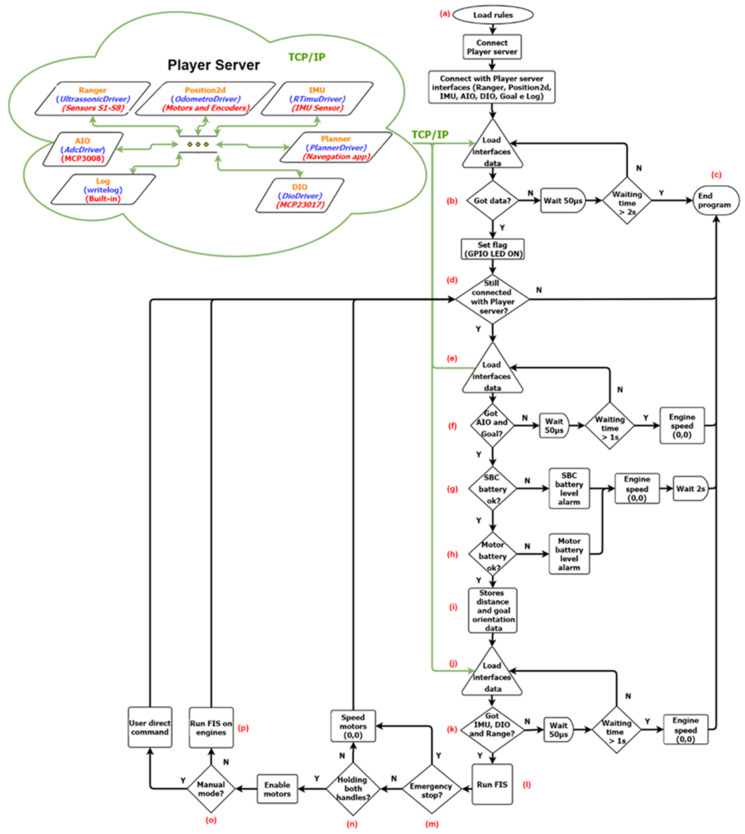
Flowchart of the main client program.

To interact with the drivers instantiated by the Player server and run the standalone navigation control algorithm, a Player server client program was created. The client program execution flowchart can be seen in Figure 12.

As shown in Figure 12, the program starts loading the FIS rule set and checks the connection to the Player server, point (a) and (b) of Figure 12. The connection to the server is checked several times during the program execution, points (b), (d), (f) and (j), as a security rule, so the data obtained from the sensors are always updated with each interaction loop. The loss of connection to the server is also a condition to close the client program, point (c), and, consequently, interrupt the power sent to the engines.

After the connection check, a new reading of the interfaces is made to verify the status of the central processing unit [40] and engine batteries, points (e), (f), (g) and (h), system interruption condition, and the information of the target distance and direction sent by the navigation application and conditionals for a nonzero FIS output, points (e), (f) and (i). With the conclusion of the previous steps, a new reading is made to obtain the updated data from the distance sensors, inertial central and contact switch, FIS inputs, point (j) and (k). If the data are obtained within the maximum time of 1 s, the FIS is executed, point (l).

For the output of the FIS to act on the engines, the emergency condition is verified, which has a direct effect on the power hardware of the engines, point (m). Two other conditions are also verified: whether the user is holding the handle of the device with both hands, point (n); and whether a manual command, point (o), is not being performed, which has priority over the FIS. Once the previous conditions are met, the FIS acts on the engines, point (p), and the client program cycle restarts with a new reading of the Player server interfaces, point (d).

### 2.4. Fuzzy Inference System

**Figure 13:**
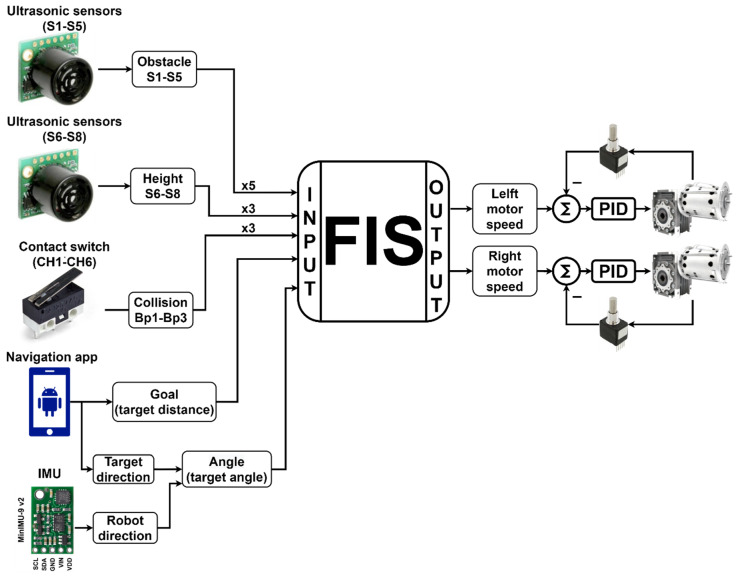
Real model fuzzy inference system (FIS) macro diagram with PID control application.

For the development of the FIS, triangular and trapezoidal membership functions were used. These forms were adopted for their simplicity and facility in writing the code. The proposed fuzzy rules were provided by an expert and refined with the observation of the interaction of the device with the virtual and real environment. The fuzzy variables used in the fuzzy inference system are related to the distance sensors, the orientation in relation to the goal, the distance to the goal, the collision condition, the desired angular velocity in the left engine, and the desired angular velocity in the right engine. Figure 13 illustrates a comprehensive FIS diagram with the sensors, actuators, and their membership functions.

The proposed FIS has 13 input variables and 2 output variables. The descriptions of these fuzzy variables used are as follows.

**Figure 14:**
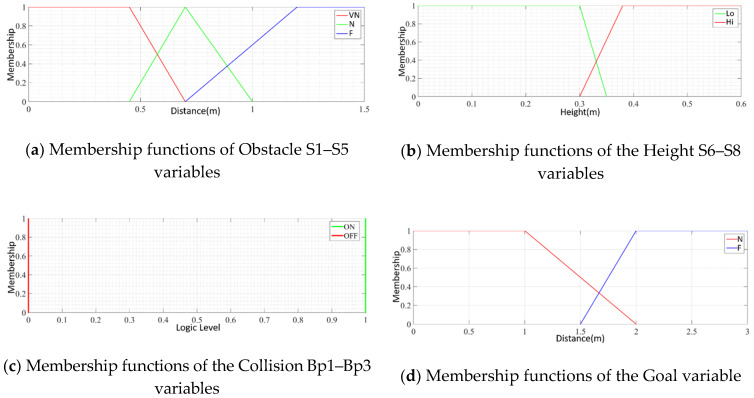

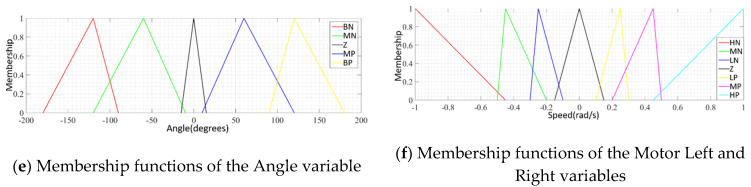
Membership functions of FIS input and output variables.

- **Obstacle**: Linguistic variables that represent the distances from the device to a collision obstacle. These variables have three membership functions each—Very Near (VN), Near (N), and Far (F)—and their universe of discourse goes from 0 to 1.5 m, as shown in Figure 14a. Altogether, five obstacle variables were used, which are associated with the S1–S5 ultrasound sensors (see Figure 2).
- **Height**: Linguistic variables that represent information on unevenness resulting from holes in the sidewalk or the presence of the curb. These fuzzy variables have two membership functions each, High (Hi) and Low (Lo), and their universe of discourse ranges from 0 to 0.6 m, as shown in Figure 14b. In total, three height variables were used, which were associated with sensors S6–S8 (see Figure 2).
- **Collision**: Linguistic variables that represent the collision of the device with some obstacle. These variables have two singleton membership functions, ON and OFF, due to the nature of the sensors that represent them, as shown in Figure 14c. These variables are associated with the contact switches CH1–CH6 (see Figure 3); however, they are separated into three groups, i.e., CH1 and CH2 represent collision variable Bp1, CH3 and CH4 the collision variable Bp2, and CH5 and CH6 the collision variable Bp3.
- **Goal**: Linguistic variable that represents the Euclidean distance of the device in relation to the goal. This variable has two membership functions, Near (N) and Far (F), and its universe of discourse ranges from 0 to 3 m, as shown in Figure 14d. The Goal variable is associated with the information about the distance to the goal, which is sent by the navigation application.
- **Angle**: Linguistic variable that represents the orientation of the device with respect to the goal. This variable has five membership functions—Big Negative (BN), Medium Negative (MN), Zero (Z), Medium Positive (MP), and Big Positive (BP)—and its universe of discourse ranges from −180° to 180°, Figure 14e. This fuzzy variable is obtained by the relationship between the direction of the objective, sent by the navigation application, and the direction of the device, obtained by the inertial centre embedded in it.
- **Motor**: Linguistic variables associated with the output variables, which represent the speed of the left and right engines. Each variable has seven membership functions—High Negative (HN), Medium Negative (MN), Low Negative (LN), Zero (Z), Low Positive (LP), Medium Positive (MP), and High Positive (HP)—represented in Figure 14f.

**Figure 15:**
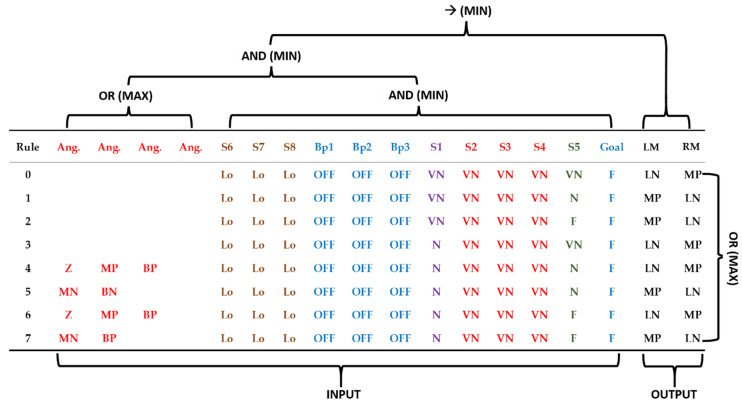
Example of application of operators for the composition of rules.

To perform the combination of the antecedents of the rules, the operators OR (Maximum) and AND (Minimum) were used. Additionally, the Minimum was used as the Mamdani inference operator and the Maximum as the aggregation operator of fuzzy rules. Figure 15 exemplifies the use of each operator in the construction and combination of the rules. Each rule has as output two fuzzy sets, which represent the individual actuation of each engine (left (LM) and right (RM) motors).

The implemented FIS uses the weighted average of the sum of the maximums as the defuzzification method, as presented in Equation (3). This method was adopted because it is easier to implement and has a better representativeness than a simple average of the maximums.

(3) $$Defuzzy_{MotorL\left(R\right)}=\frac{\sum_{i=HN}^{NP}\mu_{i}\times \;\mathrm{Average}Max_{i}}{\sum_{i=HN}^{HP}\mu_{i}}$$

To power the engines, i.e., for the FIS to have a nonzero output, the Goal variable must be different from Near (N). Additionally, there are three other conditions considered unsafe to perform autonomous navigations and, therefore, has a Zero (Z) output response from the FIS. These conditions can be observed in the last three rules presented in Table 3, which represent the following settings:

- Rule 513: Collision detection with side sensors CH1–CH2 (Bp1) and CH5–CH6 (Bp3)
- Rule 525: Detection of unevenness simultaneously in front (S7) and at the left side (S6) of the device
- Rule 526: Detection of unevenness simultaneously in front (S7) and at the right (S8) side of the device

The rationale behind the formulation of the rules was always to attempt to direct the obstacle deviation toward the goal. For this reason, the navigation and deviation rules were not separated. Another point to highlight in the methodology of creating the rules is that an attempt was made to avoid performing commands that resulted in a movement of rotation only and to avoid the negative performance of one engine being more pronounced than the positive performance of the other. The former was adopted to prevent configurations in which the robot is trapped in an oscillatory motion, whereas the second was selected so that the sum of the rules never results in a negative linear velocity, i.e., that the device never performs a reverse movement.

The output of the FIS was multiplied by a constant to scale the maximum speed of the device, so it is comfortable for autonomous navigation. During the tests, the chosen value to scale the defuzzified speed of the left and right motors is 2.6. Therefore, the maximum linear velocity reached by the device was 0.39 m/s.

To reduce the robot oscillation during some diversion manoeuvres, an external regulator was added to the set of rules. This regulator is responsible to deal with the configuration in which the robot is aligned with the objective, and its front sensors (S2–S4) indicate the object is Very Near. In this configuration, if the side sensors (S1 and S5) send the same information, the robot tends to oscillate between the negative and positive angle when the obstacle detected in front of the robot is too large. This problem occurs because, by diverting the correction of the orientation to the favourable direction, the robot starts to miss the Angle value in the opposite direction, without changing the state of the other distance sensors. The external control applied consists of monitoring the activation of the rules for this condition so that, when one of them is triggered, the symmetric rule cannot act until the robot comes into a configuration where both rules have not been activated. During this period, only the first rule to be activated is considered to compose the resulting action. The performance of this rule is the maximum activation value between symmetric rules.

The new rule base, as well as the prototype codes and diagrams, can be seen in the project repository [46].

## 3. Results and Discussion

The environment used for the tests was a sidewalk width of 3 m and curb height of 16 cm. To perform the navigation and deviation tests, five cones were inserted, spaced at approximately 1 m, in the navigation route of the sidewalk. In addition to the objects inserted, the sidewalk had a bench, totalling six obstacles that the robot could confront during its navigation. Finally, there is a gap generated by the curb of the sidewalk. Altogether, three tests were performed that differed by the position and orientation of the robot in relation to the objective, and by the direction of the goal itself, which alternated between the two directions of the sidewalk.

The test was performed within the Pontifical Catholic University of Rio de Janeiro (PUC-Rio) campus, with the navigation application in its simplified version, where the distance and orientation information are inserted manually. As shown in Section 2.3.6, this program changes the distance and orientation information to the goal through a manual command. Because of this characteristic, the distance information to the objective is not updated during the test route. In the final version of the app, this update will happen with the data from the GPS sensor of the phone installed with the Google Maps API.

In the following subsections, the route and results obtained in each of the three tests are presented in detail.

### 3.1. Route 1

**Figure 16:**
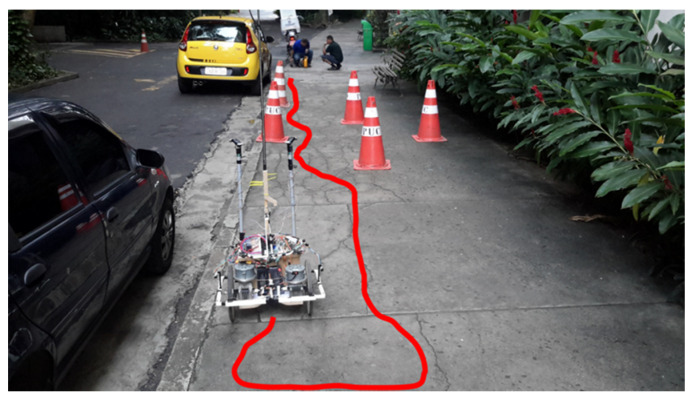
Starting position of route 1.

The first route starts with the robot at −177° from the objective, as shown in Figure 16. As mentioned in the previous section, the distance information to the objective is not updated during the test path. Therefore, the goal of the robot is only the orientation, i.e., the direction parallel to the sidewalk toward the top of Figure 16. By overcoming all obstacles, it is considered that the goal has been achieved and the test is finished. In Figure 16, traced in red, it is possible to observe the approximate path that the robot performed during route 1, and in the link [47] one can see the video of the path.

**Figure 17:**
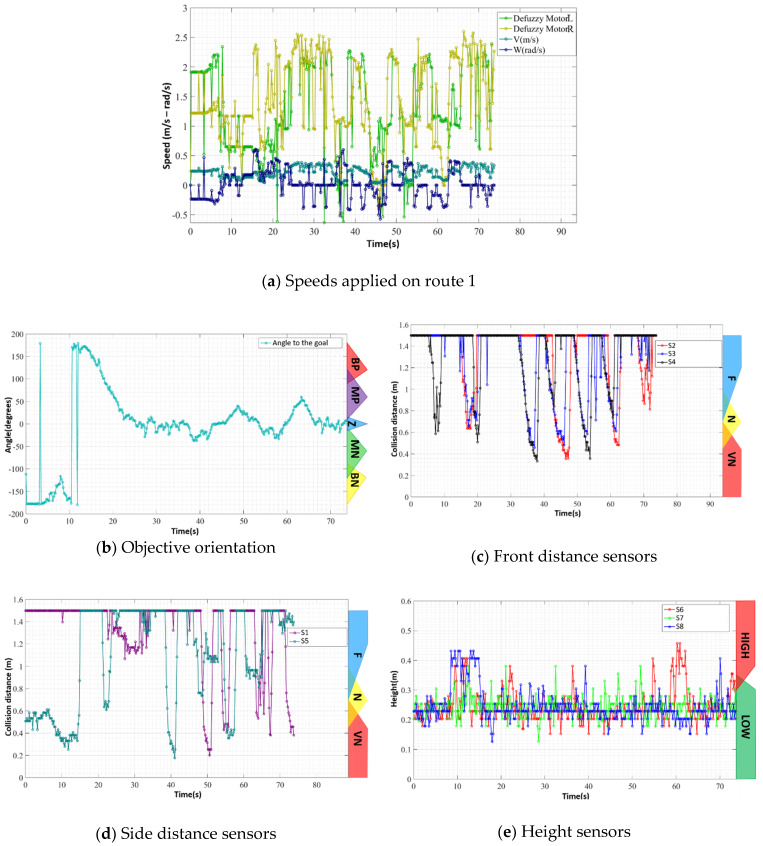
Sensor and actuator data during route 1.

Data from inputs and outputs of the control system during the execution of the test path can be observed in Figure 17. Figure 17a shows the outputs of the FIS as well as the linear and angular speeds of the device. Figure 17b shows the orientation information of the device in relation to the goal. Figure 17c provides the values of the distance sensors that measure the obstacles in front of the device—S2, S3, and S4 of Figure 2a. Figure 17d provides the information of the distance sensors that measure the obstacles on the sides of the device—S1 and S5 of Figure 2a. Finally, in Figure 17e, the information of the distance sensors that measure gap and hole obstacles is provided—S6, S7, and S8 of Figure 2. To easily compare the results of the different paths, the layout of the graphs of the inputs and outputs of the system are always displayed in this arrangement. The membership function representation is also added to the graphs of the input variables of the FIS.

The execution of this first route occurred smoothly and without large oscillations or deviations from the goal, and the device could perform the entire path without colliding. This information is shown in Figure 17a,b, which display the FIS output and orientation to the objective, respectively. In the first 10 s of the path, the device tried to align with the objective by making a right turn; however, by continuing on this route, the prototype approached an obstacle to the right, as in Figure 17c,d, and then detected the presence of the curb of the sidewalk—Figure 17e between 10 and 15 s. The prototype was forced to change its direction to reorient itself toward the objective, as in Figure 17b.

**Figure 18:**
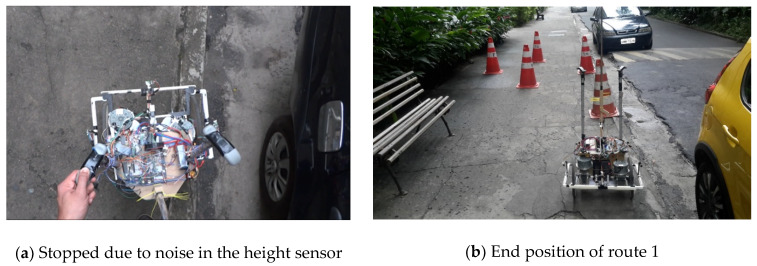
Route 1 observation points.

During the journey, the device made a small stop of less than 1 s because of a noise in the reading of S7 of Figure 2—instant *t* = 10 s of Figure 17e. This noise led it to the condition of being uneven in the front and right side. In this condition, shown in Figure 18a, the FIS provided Zero output for both motors, shown in Figure 17a at instant *t* = 10 s, because the system could not provide the necessary security for the configuration change. However, since the noise was an isolated event, the robot, after this brief stop, continued to the end of its path, finishing, as shown in Figure 18b, near the yellow car.

### 3.2. Route 2

**Figure 19:**
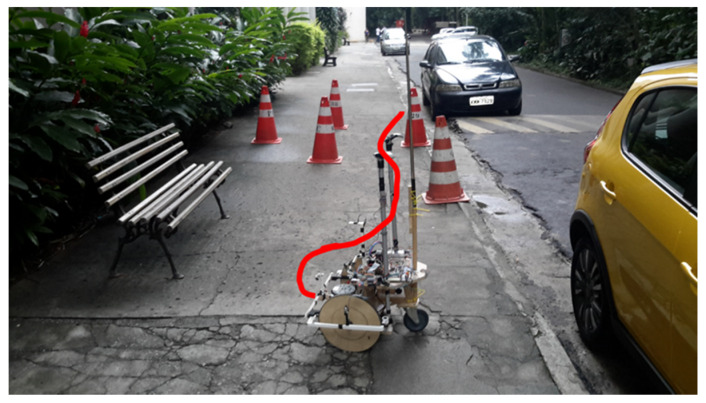
Starting position of route 2.

The second route (Figure 19) starts from a position near the end of route 1. In route 2, the initial configuration of the robot makes an angle of −90° with the objective, i.e., the direction of movement of the sidewalk, at the top of Figure 19 In the link [48], one can watch the video of route 2.

In Figure 19, as in Figure 16, an approximation of the path made by the robot is drawn in red.

**Figure 20:**
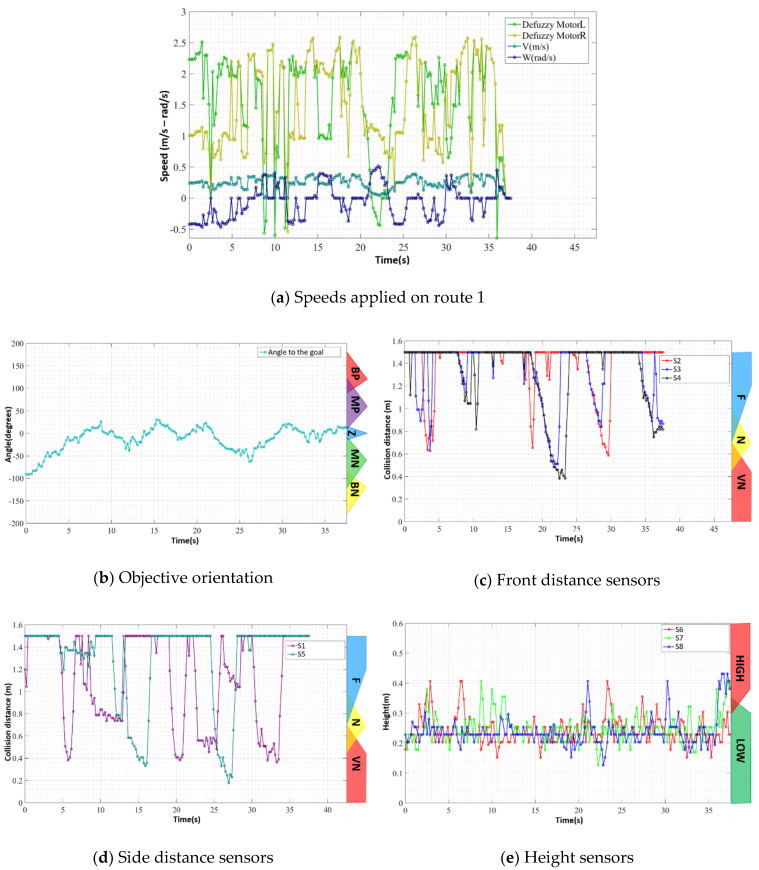
Sensor and actuator data during the route 2.

The robot, as in the previous path, performed its navigation smoothly during a good part of the route; however, a more significant oscillation was observed around the instant *t* = 11 s, as can be seen in Figure 20a. At that moment, a noise was observed, as shown in Figure 20e, in the sensor that measures the gap in front of the device—sensor S7 of Figure 20—which led the device to try to change the direction in relation to the objective. However, this command was replaced by the FIS as soon as it had the next reading.

**Figure 21:**
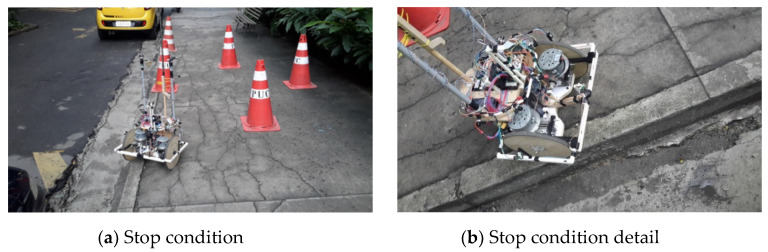
Stopped due to unsafe condition.

The rest of the path occurred without oscillations, and was directed to the objective, until, at the moment *t* = 37.5 s, after deviating from the last cone, the robot moved toward the curb, reaching an unsafe condition (Figure 21). This condition, characterised by unevenness in the front and on its right side, resulted in a Zero value for the FIS outputs—Figure 20a,e, instant *t* = 37.5 s)—requiring manual performance of the user for the engines. At this point, the test was finished, and the robot was repositioned with manual actuation. This behaviour, translated by rules 525 and 526 from Table 3, is the desired and programmed behaviour designed to increase navigation security. The data of the sensors and actuators during the performance of this route can be observed in Figure 20, with the same layout presented in Figure 17.

### 3.3. Route 3

**Figure 22:**
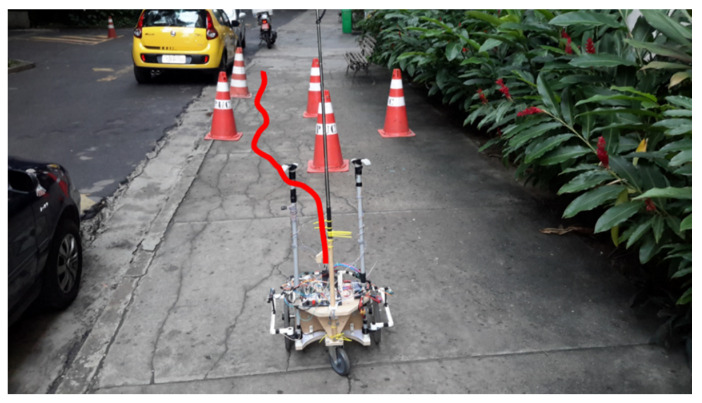
Start of route 3.

**Figure 23:**
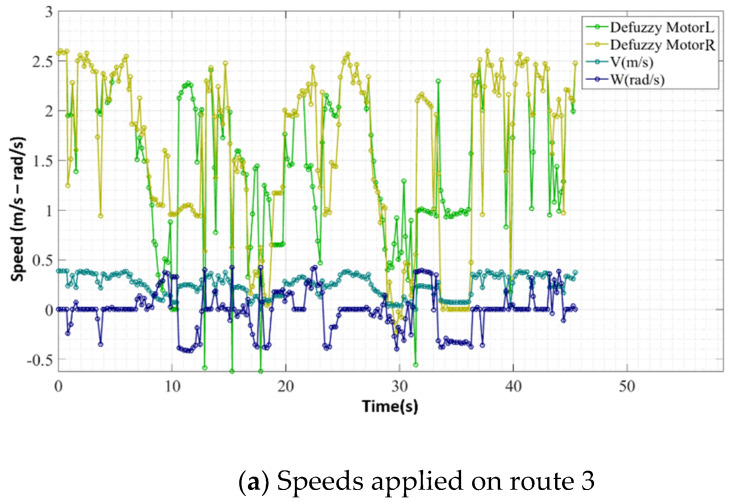

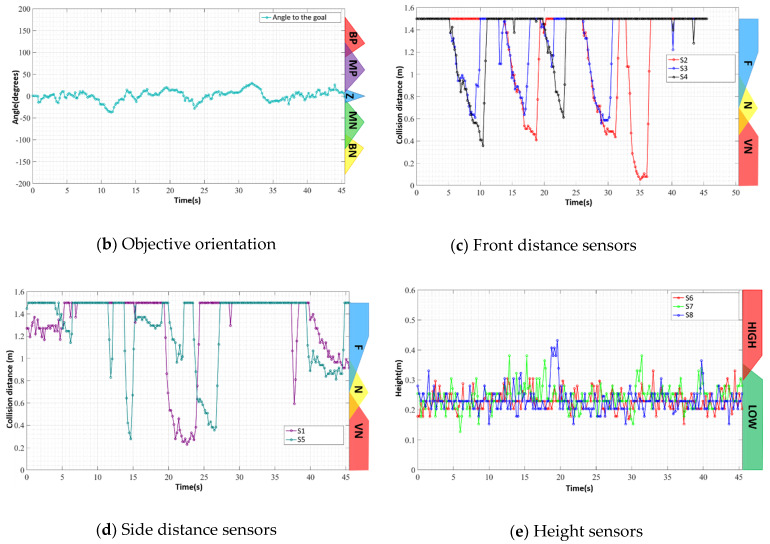
Sensor and actuator data during route 3.

The third course had the same objective as route 1; however, in this configuration, the robot started aligned with the objective and an obstacle, as shown in Figure 22. As in previous routes, the red line drawn in Figure 22 represents an approximation of the route taken during the test, and the link [49] contains the video of route 3. Figure 23 shows sensor and actuator data during route 3.

At the beginning of this route, the device approached the obstacle to remain aligned with the objective, until the rules relating to the distance to the obstacle being Very Near at sensors S3 and S4—instant *t* = 8 s of Figure 23c—were activated. As the values of these sensors began to enter the region of the “Very Near” fuzzy set, the device deviates from the obstacle—*t* > 8 s and *t* < 14 s in Figure 23a. This behaviour was expected because the device was already aligned with the objective, so the FIS trend was to slow down until it reached the bypass setting.

**Figure 24:**
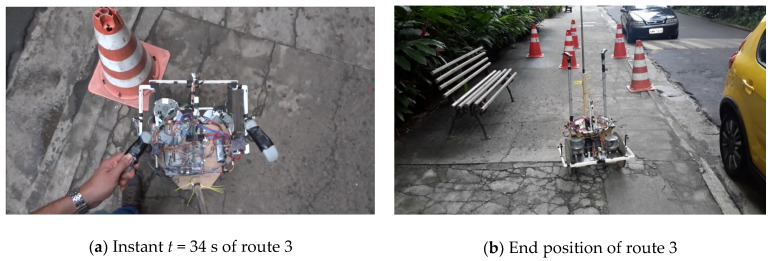
Route 3 observation points.

Another point to be observed in this route is the time interval between the moments *t* = 34 s and *t* = 37 s. In this interval, the device passed very close to an obstacle on its left side. This approach occurred because, when the device deviated from one of the obstacles, the next signalling cone was not close to the area of operation of the sensors, and it was perceived only when it was already very close to the robot, as shown in Figure 24a. Despite passing very close to the obstacle, the robot managed to bypass it, thus finishing its path.

As in the previous cases, this route was executed with gentle manoeuvres and always directed to the objective, indicating that the path travelled prioritised the frontal direction, common to the natural displacement of people. The position of the robot at the end of route 3 can be seen in Figure 24b.

### 3.4. Discussion

In the tests performed, the prototype created was able to move autonomously in an unknown environment, deviating from obstacles in a smooth way and always directed to the goal provided by the navigation application.

When faced with a configuration where the set of information provided by the sensors was not sufficient to ensure that the FIS acted safely for the user, the FIS responded with a Zero actuation. Therefore, the implemented FIS presented the desired robustness to convey the user in a safe manner. Consequently, the functionalities of assistance to locomotion, deviation from obstacles, and positioning were met. In addition to autonomous navigation, the device has mechanisms for direct user action, which always takes priority over the device automation. Direct action is essential for indoor locomotion or without navigation routes. Another feature of the displacement of the prototype is that it is only enabled, either autonomously or manually, while the user holds the handle of the cane.

The application of fuzzy logic, as a tool for the creation of the navigation algorithm, proved to be an appropriate choice. The use of fuzzy logic allowed us to create a model that can be easily interpretable due to the IF-THE set of rules. The robustness of the model made it possible to guide the user safely, performing smooth manoeuvres in an unknown environment, with the aid of a table with filters, which facilitated the verification of the system for different configurations of its fuzzy inputs and outputs. All the hardware used was integrated with the robotics Player framework. Therefore, information can be accessed by any client program that is on the same prototype network.

The observation points of the realized tests are summarized in Table 4.

## 4. Conclusions

This study aimed to develop a prototype of an autonomous robotic device to assist the locomotion of the elderly in urban environments. The main goal of this prototype was to validate and improve the navigation algorithm for the proposal task: to guide an elderly person in an urban environment autonomously. For this, the fuzzy logic model and the developed algorithm proved, based on tests results, to achieve the goal of a standalone, more-natural and safe navigation for the user and all this in an unknown and unstructured environment. Another point to highlight is that the entire prototype was developed using a cheap actuator and sensors to obtain a low-cost solution.

In terms of future work, the prototype has some points to be developed and improved. The points to be developed include the design part, structure, and the completion of the navigation application. An additional improvement is found in the inclusion of height sensors.

To improve the design, a more in-depth study on the ergonomics of the device should be carried out for the proposed task, providing a final design that is more suitable for interaction with elderly users. With the design established, the next step would be the study of the most suitable materials for the construction of the prototype, so that the final structure combines lightness and resistance.

To improve the identification of gaps, position-sensitive detector infrared sensors can be used together with ultrasound sensors. The combination of the information of these sensors of different natures could reduce the limitations of each sensor. An ultrasonic sensor operating alone proved to be an unsuitable choice for this task, because it does not provide a very large measurement horizon. When the sensor is tilted to increase its range, a large amount of noise is inserted, decreasing its accuracy. Infrared sensors, however, are sensitive to noise generated by the incidence of the sun or the low reflexibility of the contact region. Merging the data from these sensors of different natures would provide a better estimate of the uneven information.

Finally, with the updating of the structural part and the fault-monitoring system, future work must include real tests with elderly users to enable the prototype to evolve from these new results.

## 5. Patents

Karla Tereza Figueiredo Leite, Daniel de Sousa Leite, Marley Maria Bernardes Rebuzzi Vellasco, Miguel Angelo Gaspar Pinto e Frederico, Szmukler Tannenbaum. Andador Robótico Autônomo. BR Patent BR 10 2017 019713 1 issued 9 September 2017.

## Figures and Tables

**Table 1:** Compilation carried out by Rivero [19] of recommendations of scientific articles for the boundary conditions of related works.

Related Aspect	Recommendation
Speed	Speed: 0.62 m/s [26] to 1.2 m/s [27]. Cadence: 76.12 steps/min (in patients with postpolio syndrome). Ability to adapt to the maximum, minimum, and average walking speed of each user [28].
Force	The effort made by the patient should be less than 10 N [27]. The effort applied by the user should be measured, and this signal should be transformed into a speed and direction value [29].
Mass.	Less than 41 kg [27].
Project	Not obstructive to the patient’s walk. Shutdown of watch mode to enable the user to make corrections in navigation [27]. Motors on wheels or their axles for better control of the brakes to compensate for gravity on the inclined floors [29].
Cognitive assistance	Navigation capability and structured auto location in known and unknown environments [27].
Fall prevention	The device must withstand an ascent angle of 34.5° and a descent angle of 21° [27]. Sensors to detect steps well in advance [30].
Ubiquity	Shared control between device and user to correct possible device errors by “navigating” places not yet mapped or known [30].
Security with respect to locomotive deficiencies	Leakage current less than 5 mA. Gears and propellants cannot be touched [27]. Ladder and degrees are recognised, sending a notice in advance [30]. Partial support of the user weight, as well as partial support of the arms, for patients with diseases and lesions in the backbone [29]. The wheels must have a high coefficient of friction to decrease the likelihood of sliding on sloping ground [29].
Avoiding obstacles	Ability to advertise and avoid obstacles with evasive actions [27].
Obstacle warning	Sensors to announce obstacles [27,29,31].
Security with respect to visual impairments	If the sensor devices malfunction, the user or patient should be warned by audible or tactile signal [27].
Fall prevention	Ability to detect eminence fall of a person in a few seconds, given that the fall is a process of 1 or 2 s and covers various movements [31,32].
Security regarding deficiencies in balance	Maximum, minimum, and most-common values of angular turning speed, to avoid the emission of false-negative or false-positive signals [28]. Detecting the presence of the hands of the user to verify effectively whether the user suffered a fall or if it was the device that fell alone [29].

**Table 2:** Gains from proportional–integral–derivative (PID) DC motor controller.

Kp	Ki	Kd	T
2	11	0.008	0.01 s

**Table 3:** Set of rules with Zero output for both motors.

Rule	Ang.	Ang.	Ang.	Ang.	S6	S7	S8	Bp1	Bp2	Bp3	S1	S2	S3	S4	S5	Goal	LM.	RM.
415																N	Z	Z
513								ON		ON							Z	Z
525					Hi	Hi											Z	Z
526						Hi	Hi										Z	Z

**Table 4:** Summary of test observation points.

Route	Start Angle to the Goal (Degrees)	Achieved the Goal	Smooth Manoeuvres	Collided	Height Sensor Noise	Reached an Unsafe Condition
1	−177	Yes	Yes	No	Yes	No
2	−90	No	Yes	No	Yes	Yes
3	0	Yes	Yes	No	No	No
